# Radiomic Features of Acute Cerebral Hemorrhage on Non-Contrast CT Associated with Patient Survival

**DOI:** 10.3390/diagnostics14090944

**Published:** 2024-04-30

**Authors:** Saif Zaman, Fiona Dierksen, Avery Knapp, Stefan P. Haider, Gaby Abou Karam, Adnan I. Qureshi, Guido J. Falcone, Kevin N. Sheth, Seyedmehdi Payabvash

**Affiliations:** 1 Department of Internal Medicine, Yale University School of Medicine, New Haven, CT 06510, USA; 2 Department of Radiology, Yale University School of Medicine, New Haven, CT 06510, USA; 3 Independent Researcher, Guaynabo, PR 00934, USA; 4 Department of Neurology, Zeenat Qureshi Stroke Institute, University of Missouri, Columbia, MO 65211, USA; 5 Department of Neurology, Yale University School of Medicine, New Haven, CT 06510, USAkevin.sheth@yale.edu (K.N.S.)

**Keywords:** stroke, large vessel occlusion, radiomics, machine learning, collateral status

## Abstract

The mortality rate of acute intracerebral hemorrhage (ICH) can reach up to 40%. Although the radiomics of ICH have been linked to hematoma expansion and outcomes, no research to date has explored their correlation with mortality. In this study, we determined the admission non-contrast head CT radiomic correlates of survival in supratentorial ICH, using the Antihypertensive Treatment of Acute Cerebral Hemorrhage II (ATACH-II) trial dataset. We extracted 107 original radiomic features from *n* = 871 admission non-contrast head CT scans. The Cox Proportional Hazards model, Kaplan–Meier Analysis, and logistic regression were used to analyze survival. In our analysis, the “first-order energy” radiomics feature, a metric that quantifies the sum of squared voxel intensities within a region of interest in medical images, emerged as an independent predictor of higher mortality risk (Hazard Ratio of 1.64, *p* < 0.0001), alongside age, National Institutes of Health Stroke Scale (NIHSS), and baseline International Normalized Ratio (INR). Using a Receiver Operating Characteristic (ROC) analysis, “the first-order energy” was a predictor of mortality at 1-week, 1-month, and 3-month post-ICH (all *p* < 0.0001), with Area Under the Curves (AUC) of >0.67. Our findings highlight the potential role of admission CT radiomics in predicting ICH survival, specifically, a higher “first-order energy” or very bright hematomas are associated with worse survival outcomes.

## 1. Introduction

Acute intracerebral hemorrhage (ICH) remains one of the most devastating forms of stroke, accounting for a substantial proportion of stroke-related morbidity and mortality worldwide [1,2]. Despite advancements in medical and surgical interventions, its prognosis remains poor, with high early case fatality reaching 40% in population-based studies [3,4]. As the search for effective treatment for ICH continues, the rapid identification of high-risk individuals can potentially guide therapy and improve outcomes [5]. Multiple groups have identified different features of ICH shape and texture heterogeneity on admission non-contrast head CTs, which are associated with active bleeding and are a potential predictor of hematoma expansion and poor outcomes [6,7,8]. Thus, imaging features of ICH on admission head CTs—beyond hematoma volume—can provide valuable information that aids in predicting the outcome trajectory of ICH and guides clinical management decisions [9].

Radiomics represent the extension of the omics concept into the realm of medical imaging to decipher vast amounts of quantitative data from clinical scans [10]. Radiomic features, often imperceptible to the human eye, have demonstrated potential in predicting clinical outcomes in various conditions, including ICH [11,12,13]. Recent studies have shown that the radiomic features of ICH on admission CTs, representing hemorrhage texture, shape, and intensity patterns on admission non-contrast head CTs, can provide valuable prognostic information [14,15,16,17]. This is particularly important, since non-contrast head CT scans are widely available and serve as the de facto first line of imaging in patients presenting with suspected (hemorrhagic) stroke. Against this background, our study aims to prognosticate ICH survival outcomes from admission head CT radiomic features [18].

Leveraging a large dataset with detailed clinical outcome metrics from a multicentric clinical trial, we comprehensively analyzed the association between radiomic features extracted from initial non-contrast CT head scans and survival outcomes in supratentorial ICH. Then, we compared survival predictions based on the radiomic features with the “ICH Score” [19,20,21]—the most widely used clinical tool for predicting mortality in patients with spontaneous ICH. Similar to the ICH Score, a radiomics-based survival risk stratification tool can assist clinicians in making informed decisions regarding the intensity of care, potential interventions for patients with ICH, and help to facilitate goals of care discussions. Finally, we determined the predictive performance of ICH radiomic feature(s) from admission CTs for mortality at 1-week, 1-month, and 3-month follow-up intervals.

In our study, the original “first-order energy” radiomics feature emerged as the predictor of survival. This feature refers to a statistical measure that quantifies the sum of squared voxel intensities within a region of interest (ROI) in medical images. By analyzing the distribution of these intensity values, we can extract valuable information about the texture of the tissue, which may be indicative of its pathological state. For example, different areas of an image may appear lighter or darker depending on the characteristics of the tissues captured. “First-order energy” represents the distribution of voxel intensities in a region of interest, and is a way of summarizing how much ‘intensity’ is within a specific area of the image we are interested in. The “first-order energy” radiomics feature provides us a means of quantifying whether an area of interest generally has more intense (brighter) or less intense (darker) features accounting for volume, with larger lesions also having a higher “first-order energy”.

## 2. Methods

### 2.1. Patients Dataset

All clinical data and CT scans utilized in this study were from the Antihypertensive Treatment of Acute Cerebral Hemorrhage II (ATACH-II) trial, a multicentric, randomized clinical trial which evaluated antihypertensive treatment in patients with acute, spontaneous, supratentorial ICH (ClinicalTrials.gov identifier: NCT01176565) [22]. The trial compared the intensive blood pressure reduction with standard managemnet, but found no significant treatment benefit. The inclusion criteria of the trial were an age over 18 years old, the ability to receive intravenous nicardipine within 4.5 h of symptom onset, a Glasgow Coma Scale score of 5 or higher upon emergency department arrival, an International Normalized Ratio (INR) less than 1.5, and a CT scan showing an intraparenchymal hemorrhage of less than 60 cc. Additionally, subjects had to have a systolic blood pressure above 180 mmHg either at randomization or before IV antihypertensive treatment without a subsequent reduction to below 140 mmHg. The exclusion criteria included hemorrhages due to tumors, vascular malformations, aneurysms, or trauma, infratentorial hemorrhages, significant intraventricular hemorrhage, the necessity for immediate surgical intervention, current or recent pregnancy, recent use of dabigatran, a low platelet count, nicardipine sensitivity, or a pre-existing disability requiring mobility assistance [22]. Ethical compliance was ensured by the ATACH-2 investigators; our group performed post hoc analyses of anonymized data. For this study, of the 1000 trial participants, 70 had missing or corrupted baseline CT scans, missing CT data, or severe CT artifacts affecting the ICH such as motion artifacts, streak artifacts from hardware, or drain passing through or next to hematoma. Of the remaining patients, those without relevant clinical data, including survival, age, sex, race, Glasgow Coma Scale (GCS), NIH Stroke Scale score (NIHSS), baseline platelet count, baseline Activated Partial Thromboplastin Time (PTT), baseline International Normalized Ratio (INR), baseline serum glucose, systolic blood pressure prior (SBP) prior to randomization, and diastolic blood pressure (DBP) prior to randomization, were excluded from the analysis. This resulted in 871 total patients with CT imaging and relevant clinical variables included in our analyses.

### 2.2. ICH Segmentation and Extraction of Radiomic Features

Three dedicated research associates were initially trained for the manual delineation of intra-parenchymal cerebral hematomas on 100 non-contrast head CT scans. Then, each individually segmented the ICH lesions on the admission scans in a slice-by-slice fashion on the axial view, using the 3D-Slicer version 4.10.1 software, as described previously [11,12,23]. An expert neuroradiologist with over 9 years of experience (SP) conducted a thorough review and verification of all segmentations to ensure their accuracy, and made adjustments whenever necessary. Then, the non-contrast CT images and corresponding hematoma masks were preprocessed and the radiomic features of the hematomas were extracted using the pyradiomics version 3.0.1 pipeline, as detailed previously [11,12,23]. In brief, pre-processing included voxel dimension resampling to isotropic 1 mm voxels using B-spline interpolation with the hemorrhage masks restricted to a 1–200 Hounsfield unit density range. Consequently, *n* = 14 shape, *n* = 18 first-order, and *n* = 75 texture features (total *n* = 107) were extracted from the ICH lesions on the admission non-contrast head CTs (details included in Supplemental Table S1 and at (https://pyradiomics.readthedocs.io/en/latest/features.html (accessed on 10 January 2024)). Afterwards, the data were standardized by Z-scores for further statistical analysis.

### 2.3. Generation of Discovery and Validation Cohorts

To ensure the generalizability of predictive features, we randomly split the dataset into discovery (*n* = 580) and validation (*n* = 291) cohorts using the train_test_split module from the sklearn.model_selection library. We implemented stratified sampling to maintain a consistent representation of survival outcomes across datasets by preserving the ratio of deceased to surviving patients in both the discovery and independent validation cohorts.

### 2.4. Univariable Survival Analysis, Cox Proportional Hazards Model, and Kaplan–Meier Analysis

Survival was defined as the time interval from the ICH onset to either death or censoring. Censoring was applied to all patients at 3 months (2190 h or 131,400 min), as this was the conclusion of the trial follow-up period for the majority of subjects. Python version 3.10 was used for statistical analysis. A univariable survival analysis for both cox proportional hazards models and a Kaplan–Meier analysis was performed using the ‘lifelines’ version 0.28.0 python package. For the Kaplan–Meier analysis, the log-rank test, integrated within the lifelines package, was employed to compare the survival distributions of two samples. Forrest Plots were generated using the ‘matplotlib’ version 3.8.4 python package. Kaplan–Meier curves were generated using the ‘lifelines’ version 0.28.0 python package.

### 2.5. Multivariable Survival Analysis

For a multivariable survival analysis, we incorporated clinical and top original radiomic features with significant Hazard Ratios in a univariable analysis, preserving the event to variable ratio as per Vittinghoff and McCulloch [24]. To mitigate the issues of the high dimensionality and potential multicollinearity of the radiomic features, we applied the least absolute shrinkage and selection operator (LASSO) regularization (L1 ratio set to 1.0). The model’s hyperparameter, ‘alpha_min_ratio’, was set to 0.01, as per the default recommendation of the package, to adjust the strength of the L1 penalty and determine the feature significance. The LASSO Cox Proportional Hazards model was applied to the training dataset, as defined previously. After fitting the model, coefficients for each variable were extracted. To visually assess the impact of each feature, bar plots of the coefficients, the mean absolute values of the coefficients, and proportion of non-zero coefficients were generated using the ‘matplotlib’ version 3.8.4 python package. We utilized the ‘CoxnetSurvivalAnalysis’ module from the ‘sksurv’ python library for the multivariable analysis.

### 2.6. Receiver Operating Characteristics (ROC) Analysis

Survival outcomes were dichotomized based on three critical time points: 1 week, 1 month, and 3 months after ICH for both the discovery and validation cohorts. For each of the specified time points, we determined the binary outcomes by identifying patients who died within the designated time frame. Using these binary labels and the continuous values from select radiomic feature(s), we applied receiver operating characteristics (ROC) area under curve (AUC) to determine the predictive performance for mortality. To derive an optimal threshold from the ROC curve, we employed the Youden Index. From this optimal threshold, we stratified patients into ‘high-risk’ and ‘low-risk’ categories and computed the sensitivity, specificity, positive predictive value (PPV), negative predictive value (NPV), and Odds Ratio using the ‘sklearn’ version 1.4.0 library in python. Kaplan–Meier curves using the optimal threshold were also generated at previously defined timepoints for both the discovery and validation cohorts.

### 2.7. Adjusting for Multicollinearity, LASSO Logistic Regression, and Standard Logistic Regression

In the logistic regression models, we used the same variables as those used in the LASSO Cox Proportional Hazards model, however, we addressed multicollinearity by excluding the variables ‘original first=order Total Energy’ and ‘original shape Voxel Volume’, two features with high collinearity to the other features examined. LASSO logistic regression was executed using the LogisticRegression function from the sklearn.linear_model module with an L1 penalty. The solver ‘saga’ was employed, and the model was iterated 2000 times to ensure convergence. Post model fitting, the coefficients for each variable were extracted. Standard logistic regression was conducted using the Logit function from the statsmodels library. A constant term was added to the model to account for the intercept. Visual representations of the data were produced using both the ‘matplotlib’ version 3.8.4 and ‘seaborn’ version 0.12.0 python libraries.

### 2.8. Comparative Analysis of ICH Score Versus Radiomic Feature(s)

The ICH score is the most widely validated clinical-scale tool for the prognostication and prediction of mortality [20]. This 0-to-6 score is the sum of individual points from admission GCS scores of 3 to 4 (2 points) and 5 to 12 (1 point); age ≥ 80 years (1 point); infratentorial ICH (1 point); hematoma volume of ≥30 mL (1 point); and intraventricular hemorrhage (1 point) [20].

We calculated the ICH scores for all 871 patients included in the analysis. For both the ICH score and radiomic feature(s), we generated ROC curves to compare their discriminative abilities in predicting the survival outcomes at 7 days, 30 days, and 90 days post-ICH. The bootstrap method was used to compare the AUCs, employing the roc.test function from the pROC package in R.

### 2.9. Statistical Analysis of Demographic Information

For a demographics analysis, *t*-tests were used for continuous variables, the Mann–Whitney U-test was used for NIHSS and GCS, and the Chi squared test was used to compare categorical variables. A *p*-value less than 0.05 was considered as statistically significant.

## 3. Results

### 3.1. Cohort Characteristics

Table 1 compares the clinical characteristics of 58 (6.6%) patients that died during the 3-month follow-up period versus those who survived. Mortality was associated with an older age, white race, a higher NIHSS, GCS, and INR at admission, and the need for mechanical ventilation, external ventricular drainage, and surgical decompression surgery during admission. Supplemental Table S2 summarizes the clinical characteristics and hematoma volumes between the discovery (*n* = 580) and validation cohorts (*n* = 291). The discovery and validation cohorts had similar mortality rates of 39/580 (6.7%) and 19/291 (6.5%), respectively, but the validation cohort had a lower rate of White patients (*p* = 0.006) and external ventricular drainage (*p* = 0.043), as depicted in Supplemental Table S2.

### 3.2. Clinical and ICH Radiomic Features Associated with Patients’ Survival in Univariable Analysis

**Figure 1:**
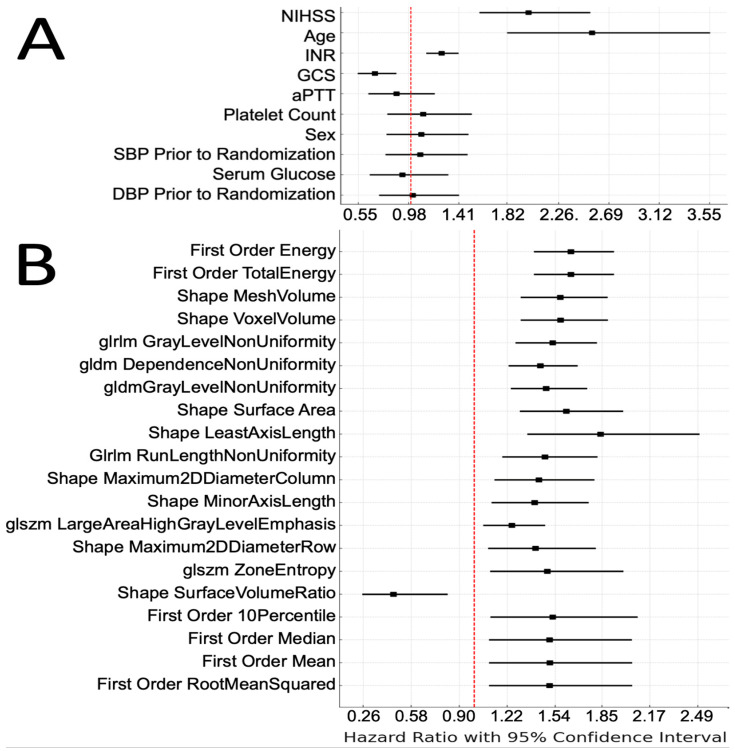
Univariate survival analysis of clinical and radiomics features. Forest plot of the hazard ratios for survival probability for (**A**) normalized clinical features and (**B**) the top 20 normalized original radiomics features determined by cox proportional hazards model. Squares denote the hazard ratio, and horizontal line represents the 95% confidence interval.

**Figure 2:**
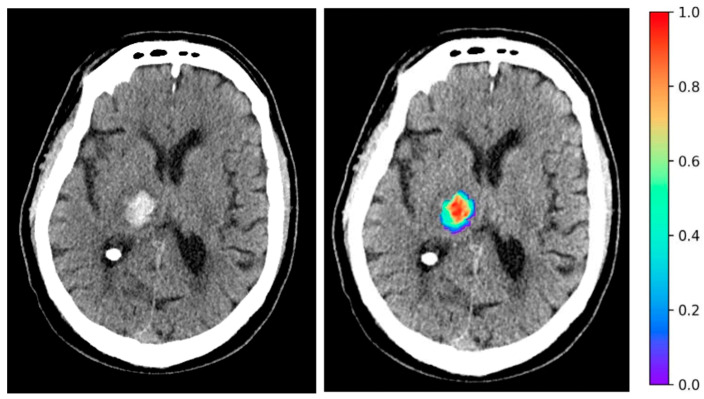
Example highlighting the ICH lesion original first-order energy radiomic feature on an axial slice of head CT scan.

To evaluate the potential associations between clinical variables and ICH radiomic features with survival outcomes, we utilized univariate Cox proportional hazards models in our discovery cohort. Figure 1A depicts the Forest plot of the hazard ratios (HRs) for significant clinical variables. An older age, higher admission NIHSS, and baseline INR were associated with higher probability of death. Conversely, a higher baseline GCS was associated with a reduced likelihood of mortality. The top 20 radiomic features of ICH on admission non-contrast head CTs which are associated with survival, ranked by their HRs, are depicted in Figure 1B. Notably, the original first-order energy, a feature that represents the sum of squared intensities (magnitude) of the voxel values inside the region of interest, was the most significant feature associated with an increased probability of death (HR = 1.64, *p* < 0.001). Figure 2 is a representative example, color-coding the original first-order energy feature of an ICH lesion on an axial slice of a head CT scan.

### 3.3. Independent Predictors of ICH Patients’ Survival

**Figure 3:**
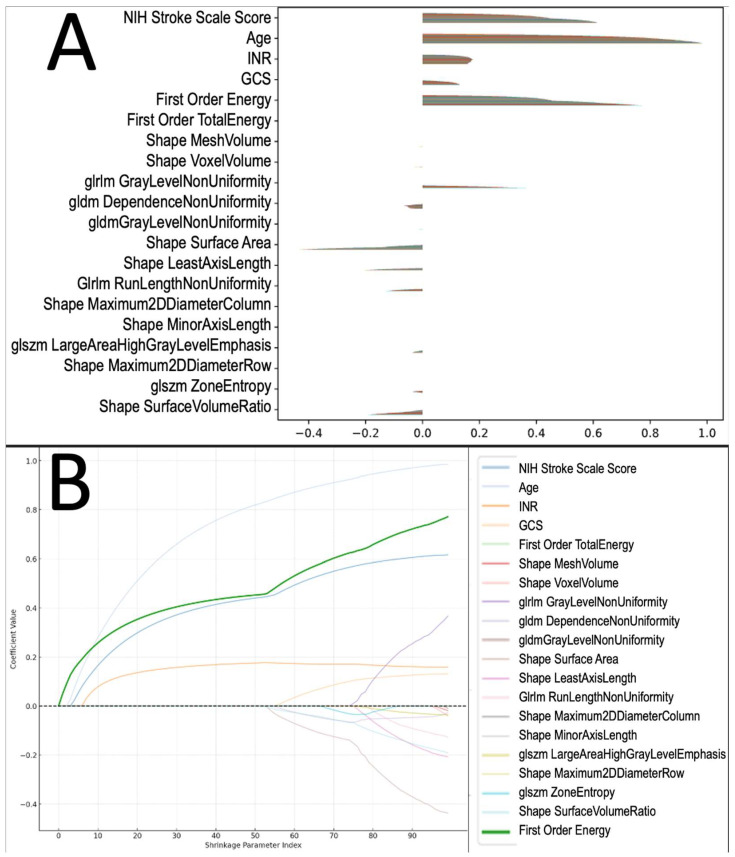
Multivariate survival analysis of clinical and radiomics features. (**A**) Lasso coefficients from multivariate Cox proportional hazards model predicting mortality. Each bar represents the coefficient value for a given feature, with the magnitude indicating the strength and direction of the association with survival. Features with positive coefficients are associated with increased risk, while those with negative coefficients are associated with decreased risk. (**B**) Coefficient paths of variables in the LASSO Cox Proportional Hazards model across different levels of regularization. Each line represents the coefficient of a specific feature as the regularization strength (denoted by the Shrinkage Parameter Index) increases. A higher Shrinkage Parameter Index indicates stronger regularization, pushing more coefficients towards zero. The color-coded paths show how each coefficient changes, with the feature “original first-order energy” represented by the thickened green line. This feature’s coefficient remains relatively stable across most levels of regularization, indicating its significance in the model compared to other variables that tend to shrink faster.

To identify the independent predictors of post-ICH survival, we applied a multivariable LASSO Cox proportional hazards model including clinical variables (age, NIHSS, INR, and GCS) and the top 16 ICH radiomic features with significant survival associations in a univariable analysis to achieve a total of 20 input variables preserving the event to variable ratio [24]. The LASSO model identified the original first-order energy, along with age, NIHSS, and baseline INR to be independently associated with an increased risk of death. A plot of the coefficients from this analysis is shown in Figure 3A. A path plot of the features included in the multivariate LASSO Cox proportional hazards model is presented in Figure 3B. A bar plot of the mean absolute coefficients and percent non-zero for each variable in the LASSO analysis is provided in Supplemental Figure S1A,B. To verify the significance of the original first-order energy parameter with regard to survival probability, Kaplan–Meier curves comparing the top quartile of patients and bottom quartile of patients with regard to original first-order energy magnitude were generated for both the discovery and validation cohorts. Increased original first-order energy was associated with a decreased survival probability in both the discovery (*p* < 0.001) and validation cohorts (*p* = 0.015) (Supplemental Figure S2A,B).

### 3.4. Survival Curves for High- and Low-Risk Patients based on the ICH Original First Order Energy

**Figure 4:**
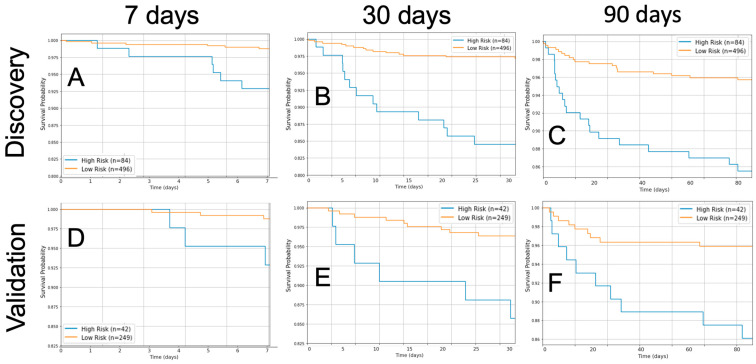
Kaplan–Meier survival analysis for the discovery and validation cohorts stratified by risk groups based on the original first-order energy feature threshold optimized for the 7-day, 30-day, and 90-day timepoints post-intial CT scan. The high-risk group (shown in blue) represents patients with values greater than or equal to the optimal threshold, whereas the low-risk group (shown in orange) represents patients with values below the threshold. (**A**) 7 days, discovery dataset (*p* < 0.001). (**B**) 7 days, validation dataset (*p* = 0.011). (**C**) 30 days, discovery dataset (*p* < 0.001). (**D**) 30 days, validation dataset (*p* = 0.003). (**E**) 90 days, discovery dataset (*p* < 0.001). (**F**) 90 days, validation dataset (*p* = 0.003).

We applied a Kaplan–Meier analysis providing a focused evaluation within each distinct time frame comparing high- versus low-risk patients based on Youden Analysis dichotomization. Adjusted events were considered, ensuring a more accurate representation of the survival probability over the specified intervals. For each time point, this analysis was conducted on both the discovery and validation cohorts. The analysis revealed that high-risk patients with high original first-order energy consistently had a decreased survival probability (*p* < 0.05 in all cases) (Figure 4A–F).

### 3.5. The ICH Original First-Order Energy Feature Predicting Death at 1 Week, 1 Month, and 3 Months

**Figure 5:**
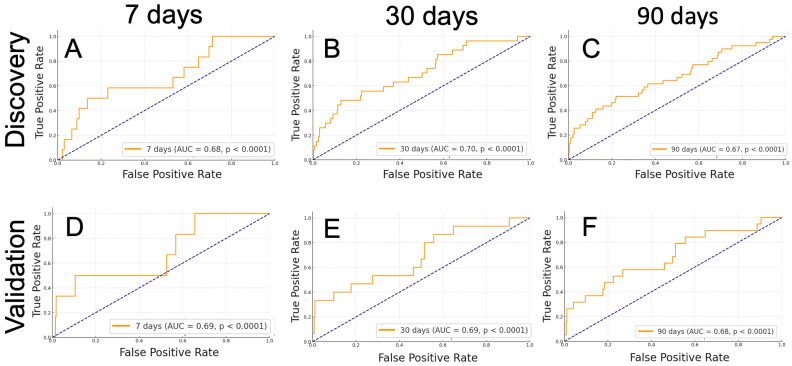
ROC curves for the prediction of mortality using the ICH “original first-order energy” as a feature at the following time points post-CT scan in both the discovery and validation cohorts. The ROC curve is compared against the diagonal dashed line, which represents random guessing. (**A**) 7 days, discovery dataset (AUC = 0.68, *p* < 0.0001). (**B**) 30 days, discovery dataset (AUC = 0.70, *p* < 0.0001). (**C**) 90 days, discovery dataset (AUC = 0.67, *p* < 0.0001). (**D**) 7 days, validation dataset (AUC = 0.69, *p* < 0.0001). (**E**) 30 days, validation dataset (AUC = 0.69, *p* < 0.0001). (**F**) 90 days, validation dataset (AUC = 0.68, *p* < 0.0001).

To further validate the results associated with the original first-order energy feature, we conducted an ROC analysis at 1-week, 1-month, and 3-month post-ICH intervals. In the discovery cohort, the ICH “original first-order energy” could predict mortality at 1 week (AUC = 0.68, *p* < 0.0001), 1 month (AUC = 0.70, *p* < 0.0001), and 3 months (AUC = 0.67, *p* < 0.0001) post-ICH (Figure 5A–C). Applying a Youden analysis to ascertain the optimal threshold for the prediction of death at each follow-up interval in the discovery cohort, at the optimal cutoff point, the ICH “original first-order energy” was associated with death Odds Ratio (OR) = 6.14 at 1-week (*p* = 0.019), OR = 6.14 at 1-month (*p* < 0.0001), and OR = 3.66 at 3-month (*p* = 0.0006) follow-up. Similarly, in the validation cohort, the ICH “original first-order energy” could predict mortality at 1 week (AUC = 0.69, *p* < 0.0001), 1 month (AUC = 0.69, *p* < 0.0001), and 3 months (AUC = 0.68, *p* < 0.0001) post-ICH (Figure 5D–F).

### 3.6. Comparing the ICH Score with the ICH Original First-Order Energy Feature in Predicting Mortality

We also compared the ICH score with the ICH original first-order energy in the prediction of mortality at 1-week, 1-month, and 3-month follow-ups in the whole dataset. For the 1-week mortality outcome, the ICH score achieved an AUC = 0.612 compared with the ICH original first-order energy AUC = 0.699 (*p* = 0.069). For the 1-month mortality outcome, the ICH score achieved an AUC = 0.612 compared with the ICH original first-order energy AUC = 0.6987 (*p* = 0.079). For the 3-month mortality outcome, the ICH score achieved an AUC = 0.591 compared with the ICH original first-order energy AUC = 0.675 (*p* = 0.072).

## 4. Discussion

We showed that the radiomic features of hematoma on admission non-contrast head CTs can predict 1-week to 3-month ICH mortality, almost more accurately than the widely used multifactorial ICH score (comparison *p* values in 0.06 to 0.08 range). Using detailed and prospectively collected outcome measures from the multicentric ATACH-2 trial, we found that the hematoma “original first-order energy” radiomic feature provides unique survival prognostication in patients with supratentorial ICH presenting with a systolic blood pressure >180 mmHg. The “original first-order energy” represents the magnitude of the voxel signal intensity values confounded with the lesion volume [25]—i.e., brighter and larger hematomas on head CTs have higher original first-order energy (Figure 2). In our univariable and multivariable analyses, this radiomic feature had a stronger association with survival outcome than ICH volume and was an independent predictor of survival alongside age, NIHSS, and INR. This highlights the prognostic implication of hematoma texture on admission head CTs, and the potential role of automatically extracted radiomic features in ICH risk stratification. The early identification of patients at a high risk for poor outcomes can guide informed discussions about prognosis with patients’ families, setting long-term goals of care, decisions regarding invasive procedures, and intensive monitoring. Given the time-sensitive nature of ICH, The original first-order energy feature, which can be automatically extracted from routine admission head CT scans, provides an objective measure for rapidly risk stratifying patients in the acute phase after ICH. Beyond prognostication, integrating this radiomic biomarker into clinical workflows could assist physicians in making time-sensitive decisions regarding the intensity of monitoring, aggressiveness of interventions, and goals of care discussions with patients’ families. For example, our results from Table 1 indicate that surgical decompression may be a negative prognostic indicator, which may be representative of the worse clinical status of a patient requiring surgical decompression; the energy feature highlighted in this study could provide an additional datapoint to help guide a patient towards or away from surgical decompression. Practical implementation would involve developing automated pipelines that segment ICH on admission CT scans and extract the energy feature value, which can then be incorporated into electronic medical-record-integrated clinical decision support tools to help guide patient management.

Our study is the first to demonstrate the 3-month survival correlates of admission head CT hematoma radiomics. With regard to radiomics applications in clinical outcome prediction, prior studies have demonstrated the potential of radiomics in predicting hematoma expansion or clinical outcomes in ICH patients [14,15,16,17]. Our study is the first to specifically focus on the survival correlates of ICH radiomics in 1-week to 3-month periods, and demonstrates the utility of a single radiomics parameter for survival prognostication independent of other clinical risk factors. Our results, along with prior studies, confirm the potential role of ICH radiomics from admission non-contrast head CTs in risk stratification and treatment guidance.

Several predictive scoring scales have been developed for ICH prognostication; ref. [8,19,20,26] yet, the “ICH score”, which was originally devised for the prediction of 30-day mortality, remains the most widely validated and used clinical-scale tool for ICH prognostication [20]. Although the ICH score was primarily developed as a clinical grading scale and a communication tool between stroke centers, its application has been expanded for outcome prediction by the American Heart Association and the Joint Commission for stroke centers [27]. This underscores the need for prognostic tools to guide the course of patient care. In our results, a single radiomic feature of hematoma on admission head CT provided survival prognostic information similar (and almost better than) to the multifaceted ICH score for 1-week to 3-month mortality. With recent advances in the automated segmentation of hematoma on non-contrast CT imaging, a fully automated pipeline can identify, delineate, and extract the radiomic features of ICH from baseline head CT imaging to provide survival predictions immediately after the admission scan.

Our research underscores the utility of radiomics not merely as a tool for predicting patient survival, but importantly, for refining treatment goals in the context of ICH. The ICH score, traditionally used to estimate mortality risk, serves as a guide for clinicians to set appropriate treatment objectives [27]. Our study’s use of radiomic features extends this concept by rapidly facilitating the identification of high-risk patients, potentially assisting in the decision-making process to avoid overly aggressive treatments that may not improve quality of life or survival. This approach respects the delicate balance between intervention and quality of care, aiming to prevent overtreatment and instead promote tailored therapeutic strategies that align with individual prognosis and patient-centered care goals.

Energy is a measure of the magnitude of the voxel intensity values in an image, as well as the size of the lesion; thus, a brighter and/or larger hematoma has higher “first-order energy” [25]. Thus, higher energy may represent active hemorrhage in larger hematomas. Notably, the ICH energy was not among those directly associated with 3-month outcomes (measured by modified Rankin Score) or hematoma expansion reported in prior studies [11,12]. As such, higher first-order energy feature values of acute supra-tentorial ICH on admission CT are distinctively predictive of a higher mortality risk among these patients.

Of note, the pyradiomics pipeline allows for the extraction of additional features from eight decompositions per original image after applying “coif-1” high- versus low-wavelet transform, as well as “edge-enhancement” Laplacian of Gaussian filters [25]. Hence, the total number of radiomic features extracted from each target lesion can increase to over a thousand [10,11,12]. However, the issue of collinearity between radiomic features intensifies with the addition of wavelet and edge-enhancement derivatives. This, in turn, mandates the incorporation of extra feature selection steps within the analytical process. In our analysis, due to the outcome imbalance and relatively small (~7%) mortality rate, we chose to limit the analysis to features extracted from the original scans—thus referred to as the “original” first-order energy feature. The LASSO multivariable analysis was adept at addressing the issue of collinearity among the 107 original features included in our analysis.

The main strength of our study is its use of a large multicentric cohort with detailed prospective follow-up information. However, our study is subject to limitations that warrant further discussion. Our study acknowledges several limitations that must be considered when interpreting the results. Primarily, the cohort analyzed was restricted by the inclusion criteria and patient characteristics of the ATACH-2 trial, which may impact the generalizability of our findings to broader ICH patient demographics. Specifically, in comparison to the broader ICH patient demographic, our cohort demonstrated a lower motility rate, a discrepancy that is likely attributable to the smaller hematoma volumes within the ATACH-2 trial, directly resulting from the trial’s inclusion criteria for an admission ICH volume of less than 60 mL for enrollment. Moreover, the ATACH-2 trial’s design, focusing on a specific subset of ICH patients with attributes such as supratentorial ICH with a systolic blood pressure above 180 [22], inherently narrows the scope of our investigation. Consequently, the applicability of our conclusions to ICH patients, especially those with larger hematoma, infratentorial hemorrhage, and non-hypertensive mechanisms such as cerebral amyloid angiopathy, may be limited. Additionally, given the limitations of the data available from the ATACH-2 trial, we were limited to only a 3-month follow up for the patients in our study; whereas, the long-term prognostic outcome of ICH patients is more accurately represented at the one-year post-ICH mark [28], underscoring the need for analyses of datasets with more extended follow-up durations [28]. As such, the prognostic value of the radiomic features studied are unclear beyond 3 months. Moreover, while our analysis concentrated on the original radiomic features presented in the manuscript, the findings suggest that future research should investigate additional radiomic features, such as wavelet-transformed or edge-enhanced features, to potentially uncover further prognostic information. Larger cohorts may also facilitate the inclusion of features extracted from derivative images after applying wavelet and edge-enhancement filters.

## 5. Conclusions

Using a large multicentric cohort of patients with acute supra-tentorial ICH, we showed that radiomic features extracted from hematomas on admission non-contrast head CT imaging can predict survival in the 1-week to 3-month follow-up period. Specifically, we found that the higher first-order energy feature of ICH, which represents brighter, larger hematomas, is associated with a higher risk of mortality. Along with age, NIHSS, GCS, and INR, this feature was an independent predictor of mortality in both the discovery and validation cohorts. Of note, this single radiomic feature provides survival predictive information similar to (and almost better than) the multifactorial ICH score, which has been the most widely used clinical scale for ICH prognostication. Although our analysis is limited by the selection criteria of the ATACH-2 trial, our study was the first to show the capability of admission hematoma radiomics to predict the 3-month survival post-ICH period. Early prediction of survival can inform prognosis discussions with patients’ family members, guide treatment decisions, and set goals of care for ICH patients.

## Figures and Tables

**Table 1:** The baseline demographics, clinical findings, and laboratory results as well as interventions during the admission categorized between alive versus deceased.

	Alive	Deceased	*p*-Value
Age (years)	61.43 ± 12.57	71.28 ± 14.18	<0.001
Sex (Male)	509 (62.61%)	32 (55.17%)	0.323
Race: Black or African-American	104 (12.79%)	7 (12.07%)	1
Race: White	200 (24.60%)	38 (65.52%)	<0.001
Race: Other	3 (0.37%)	1 (1.72%)	0.639
Race: Unknown/Not reported	6 (0.74%)	2 (3.45%)	0.168
Race: Asian	498 (61.25%)	10 (17.24%)	<0.001
Race: American Indian or Alaska Native	3 (0.37%)	0 (0.00%)	1
Systolic blood pressure (mm Hg)	175.01 ± 24.15	177.07 ± 31.81	0.541
Diastolic blood pressure (mm Hg)	94.22 ± 19.11	95 ± 29.82	0.773
NIH Stroke Scale score	10 (9)	16.5 (8.75)	<0.001
Glasgow Coma Scale (GCS) score	15 (1)	14 (4)	<0.001
Platelet count (×10^3^/mm^3^)	221.16 ± 61.00	225.79 ± 83.90	0.587
Activated partial thromboplastin time (s)	27.60 ± 5.72	26.17 ± 7.73	0.072
International Normalaized Radio (INR)	0.9863 ± 0.1377	1.1086 ± 0.2799	<0.001
Serum glucose (mg/dL)	138.53 ± 55.26	138.46 ± 49.48	0.9927
Mechanical ventilation	73 (8.98%)	34 (58.62%)	<0.001
External ventricular drainage	38 (4.67%)	17 (29.31%)	<0.001
Surgical evacuation decompression	29 (3.57%)	7 (12.07%)	0.005

The values are presented as mean ± standard deviation, median (interquartile), or frequency (percentage), for continuous, ordinal, and categorical variables, respectively.

## Data Availability

All data are available from the corresponding author upon reasonable request.

## Supplementary Materials

The following supporting information can be downloaded at: https://www.mdpi.com/article/10.3390/diagnostics14090944/s1, Figure S1: A bar plot of the (A) mean absolute coefficients and (B) percent non-zero for each variable in the LASSO cox analysis. The Original first order Energy feature consistently demonstrates high mean absolute coefficients and proportion of non-zero coefficients across the model iterations, signifying its importance in the analysis; Figure S2: Kaplan-Meier survival analysis of the ICH original first order Energy. (A) Survival outcomes associated with varying original first order Energy in the training dataset. Top quartile of patients (*n* = 145, blue) compared to the bottom quartile of patients (*n* = 145, orange). Log rank comparison *p*-value = 0.0006. (B) Survival outcomes associated with varying original first order Energy in the test dataset. Top quartile of patients (*n* = 73, blue) compared to the bottom quartile of patients (*n* = 73, orange). Log rank comparison *p*-value = 0.0157; Table S1: List of original radiomic features; Table S2: The baseline demographics, clinical findings, and laboratory results as well as interventions during the admission categorized between discovery versus validation cohorts.
