# Supplementary material for: Radiomic Features of Acute Cerebral Hemorrhage on Non-Contrast CT Associated with Patient Survival
Source: Diagnostics (Basel). 2024 Apr 30;14(9):944. doi: 10.3390/diagnostics14090944 (PMC11083693; doi:10.3390/diagnostics14090944)
Supplement: Supplementary file 1 [file diagnostics-14-00944-s001.zip › diagnostics-2959692-supplementary.pdf]

Supplementary Table S1. List of original radiomic features

Feature Family		Feature name
**First-order**	1	10th percentile
	2	90th percentile
	3	Energy
	4	Entropy
	5	Interquartile Range
	6	Kurtosis
	7	Maximum
	8	Mean
	9	Mean Absolute Deviation
	10	Median
	11	Minimum
	12	Range
	13	Robust Mean Absolute Deviation
	14	Root Mean Squared
	15	Skewness
	16	Total Energy
	17	Uniformity
	18	Variance
**Shape**	1	Elongation
	2	Flatness
	3	Least Axis Length
	4	Major Axis Length
	5	Maximum 2D Diameter Column
	6	Maximum 2D Diameter Row
	7	Maximum 2D Diameter Slice
	8	Maximum 3D Diameter
	9	Mesh Volume
	10	Minor Axis Length
	11	Sphericity
	12	Surface Area
	13	Surface Area to Volume Ratio
	14	Voxel Volume
**Texture - Gray Level Cooccurrence Matrix Features (glcm)**	1	Autocorrelation
	2	Cluster Prominence
	3	Cluster Shade
	4	Cluster Tendency
	5	Contrast

	6	Correlation
	7	Difference Average
	8	Difference Entropy
	9	Difference Variance
	10	Informational Measure of Correlation 1
	11	Informational Measure of Correlation 2
	12	Inverse Difference
	13	Inverse Difference Moment
	14	Inverse Difference Moment Normalized
	15	Inverse Difference Normalized
	16	Inverse Variance
	17	Joint Average
	18	Joint Energy
	19	Joint Entropy
	20	Maximal Correlation Coefficient
	21	Maximum Probability
	22	Sum Average
	23	Sum Entropy
	24	Sum of Squares
**Texture - Gray Level Size Zone Matrix Features (glszm)**	1	Gray Level Non-Uniformity
	2	Gray Level Non-Uniformity Normalized
	3	Gray Level Variance
	4	High Gray Level Zone Emphasis
	5	Large Area Emphasis
	6	Large Area High Gray Level Emphasis
	7	Large Area Low Gray Level Emphasis
	8	Low Gray Level Zone Emphasis
	9	Size Zone Non-Uniformity
	10	Size Zone Non-Uniformity Normalized
	11	Small Area Emphasis
	12	Small Area High Gray Level Emphasis
	13	Small Area Low Gray Level Emphasis
	14	Zone Entropy
	15	Zone Percentage
	16	Zone Variance
**Texture - Gray Level Run Length Matrix Features (glrlm)**	1	Gray Level Non-Uniformity
	2	Gray Level Non-Uniformity Normalized
	3	Gray Level Variance
	4	High Gray Level Run Emphasis

	5	Long Run Emphasis
	6	Long Run High Gray Level Emphasis
	7	Long Run Low Gray Level Emphasis
	8	Low Gray Level Run Emphasis
	9	Run Entropy
	10	Run Length Non-Uniformity
	11	Run Length Non-Uniformity Normalized
	12	Run Percentage
	13	Run Variance
	14	Short Run Emphasis
	15	Short Run High Gray Level Emphasis
	16	Short Run Low Gray Level Emphasis
**Texture - Neighboring Gray Tone Difference Matrix Features (ngtdm)**	1	Busyness
	2	Coarseness
	3	Complexity
	4	Contrast
	5	Strength
**Texture - Gray Level Dependence Matrix Features (gldm)**	1	Dependence Entropy
	2	Dependence Non-Uniformity
	3	Dependence Non-Uniformity Normalized
	4	Dependence Variance
	5	Gray Level Non-Uniformity
	6	Gray Level Variance
	7	High Gray Level Emphasis
	8	Large Dependence Emphasis
	9	Large Dependence High Gray Level Emphasis
	10	Large Dependence Low Gray Level Emphasis
	11	Low Gray Level Emphasis
	12	Small Dependence Emphasis
	13	Small Dependence High Gray Level Emphasis
	14	Small Dependence Low Gray Level Emphasis

Adapted from Haider SP, et al. (Front Neurosci.;17:1225342)

Supplementary Table S2. The baseline demographics, clinical findings, and laboratory results as well as interventions during the admission categorized between discovery versus validation cohorts.

	Discovery	Validation	P-value
Age (years)	61.82 ± 12.74	62.62 ± 13.26	0.395
Sex (Male)	352 (60.7%)	189 (64.9%)	0.251
Race: Black or African-American	69 (11.9%)	42 (14.4%)	0.342
Race: White	176 (30.3%)	62 (21.3%)	0.006
Race: Other	2 (0.34%)	2 (0.69%)	0.862
Race: Unknown / Not reported	2 (0.34%)	6 (2.06%)	0.033
Race: Asian	330 (56.9%)	178 (61.1%)	0.257
Race: American Indian or Alaska Native	1 (0.17%)	2 (0.69%)	0.542
Systolic blood pressure (mm Hg)	175.40 ± 24.65	174.66 ± 24.89	0.677
Diastolic blood pressure (mm Hg)	94.08 ± 19.90	94.65 ± 20.15	0.691
NIH Stroke Scale score	11.0 (10.0)	10.0 (10.0)	0.465
Glasgow Coma Scale (GCS) score	15.0 (2.0)	15.0 (2.0)	0.967
Platelet count (x 10^3 / mm^3)	222.45 ± 60.30	219.51 ± 67.38	0.529
Activated partial thromboplastin time (sec)	27.35 ± 6.00	27.81 ± 5.63	0.270
International normalized ratio (INR)	1.00 ± 0.17	0.99 ± 0.10	0.353
Serum glucose (mg/dL)	138.80 ± 56.28	137.98 ± 52.02	0.830
Intracerebral hemorrhage volume (mL)	13.3 ± 12.3	13.9 ± 11.9	0.417
Mechanical ventilation	77 (13.4%)	30 (10.4%)	0.254
External ventricular catheter	44 (7.6%)	11 (3.8%)	0.043
Surgical evacuation decompression	27 (4.7%)	9 (3.1%)	0.364

The values are presented as mean ± standard deviation, median (interquartile), or frequency (per-centage), for continuous, ordinal, and categorical variables, respectively

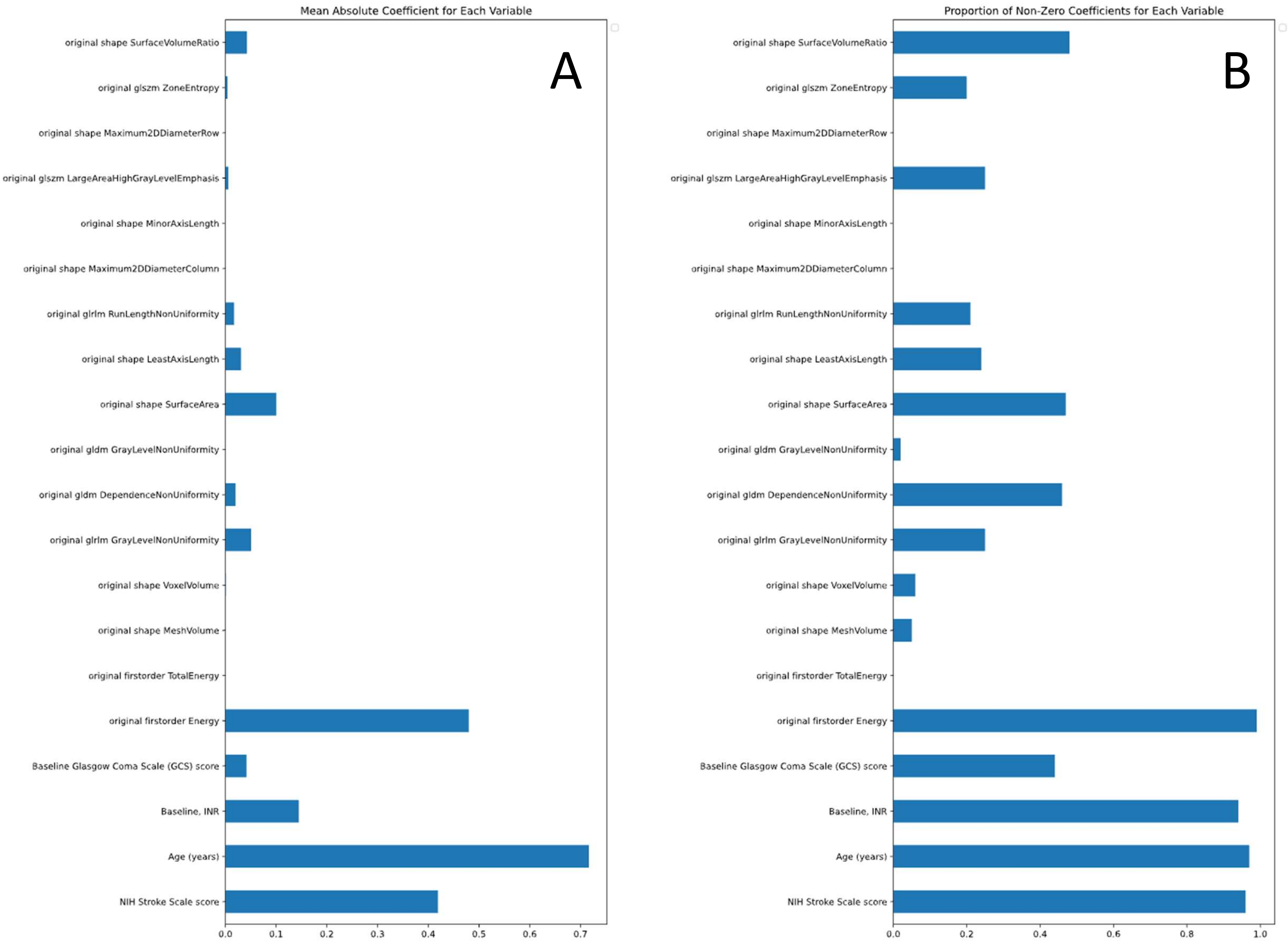

Supplementary Figure S1. A bar plot of the (A) mean absolute coefficients and (B) percent non-zero for each variable in the LASSO cox analysis. The Original first order Energy feature consistently demonstrates high mean absolute coefficients and proportion of non-zero coefficients across the model iterations, signifying its importance in the analysis.

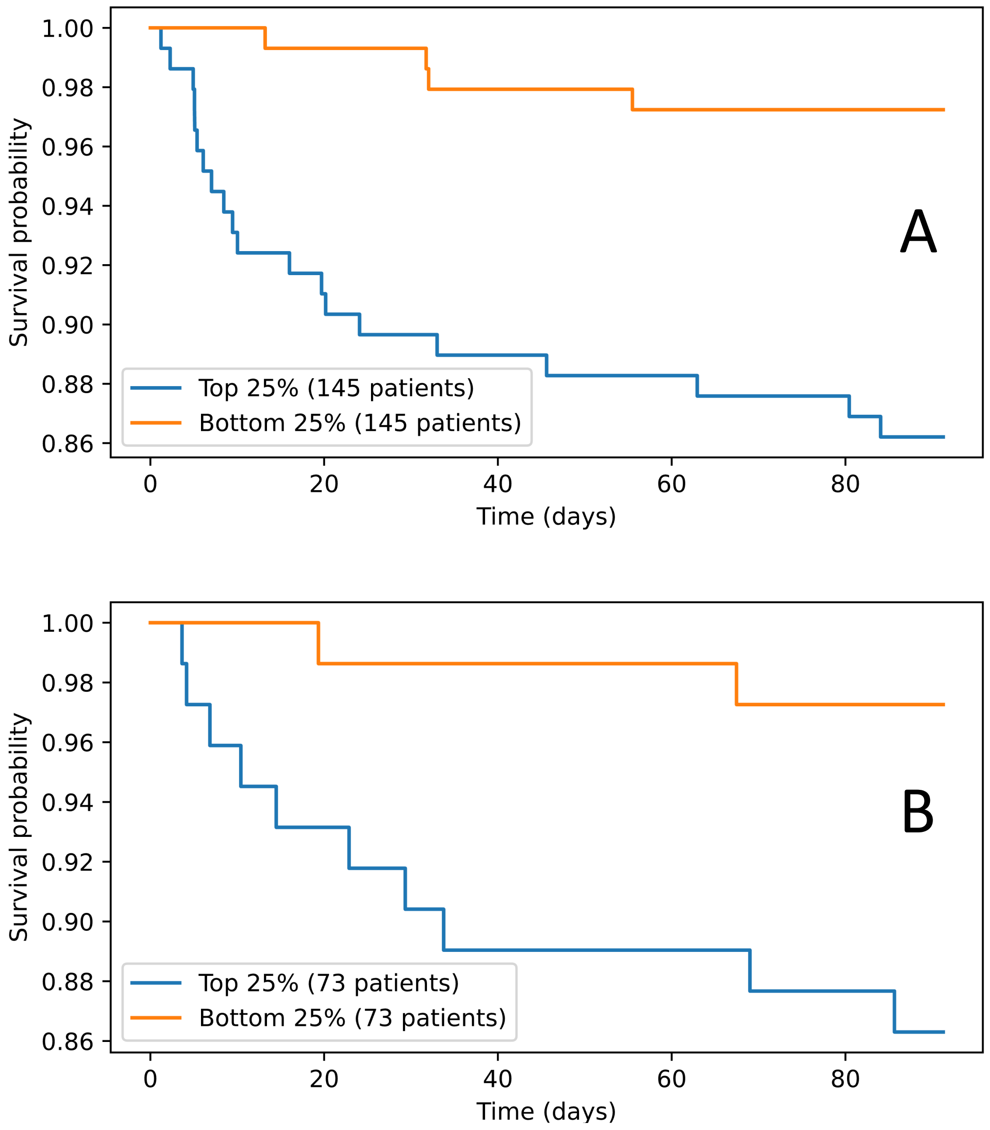

Supplementary Figure S2. Kaplan-Meier survival analysis of the ICH original first order Energy**.** (A) Survival outcomes associated with varying original first order Energy in the training dataset. Top quartile of patients (n = 145, blue) compared to the bottom quartile of patients (n = 145, orange). Log rank comparison p-value = 0.0006. (B) Survival outcomes associated with varying original first order Energy in the test dataset. Top quartile of patients (n = 73, blue) compared to the bottom quartile of patients (n = 73, orange). Log rank comparison p-value = 0.0157.
